# Feasibility and Process Evaluation of a Screening Intervention for Early Detection of Malnutrition Risk Among Community-Dwelling Older Adults Receiving Home Care Services

**DOI:** 10.3390/geriatrics11040112

**Published:** 2026-08-21

**Authors:** Tine Louise Launholt, Palle Larsen, Jemma Hawkins, Hanne Kaae Kristensen

**Affiliations:** 1 Department of Nursing, UCL University College, DK-7100 Vejle, Denmark; 2 Department of Clinical Research, University of Southern Denmark, DK-5000 Odense, Denmark; 3 Health Sciences Research Centre, UCL University College, DK-5000 Odense, Denmark; pala@ucl.dk; 4 Centre for Development, Evaluation, Complexity and Implementation in Public Health Improvement (DECIPHer), Cardiff University, Cardiff CF10 3AT, UK; 5 Research Unit for CIMT—Center for Innovative Medical Technology, Odense University Hospital, DK-5000 Odense, Denmark

**Keywords:** geriatrics, malnutrition, community-dwelling older adults, home care, screening, complex intervention, feasibility, process evaluation

## Abstract

Background: Malnutrition is prevalent among older adults (OAs), ≥65 years, receiving home care services. Despite recommendations for early detection and multifactorial interventions, a gap remains between routine practice and evidence. We therefore developed a complex intervention for early detection of malnutrition risk in a Danish home care setting, comprising an implementation strategy with staff learning activities and a screening procedure with a start-up conversation and follow-up screenings. This article outlines the feasibility test and process evaluation of the intervention. Methods: Guided by the Medical Research Council (MRC/NIHR) framework for complex interventions and MRC process evaluation guidance, the evaluation was conducted in two parts. Part 1 focused on case-level feasibility, while Part 2 examined implementation feasibility within routine home care practice. Qualitative data were collected through participant observations, informal interviews, semi-structured individual interviews, focus group interviews, and a participatory workshop. Quantitative data were extracted from the electronic patient record (NEXUS, Systematic A/S, Aarhus, Denmark). Qualitative data were analysed using qualitative content analysis, and quantitative data using descriptive statistics. Results: All eligible OAs (12/12) received a start-up conversation in Part 1 compared with 5/16 (31%) in Part 2, while follow-up screenings declined from 61/71 (86%) to 37/50 (74%). Overall, staff perceived the learning activities as useful and were able to deliver the screening procedure. However, delivery and implementation were influenced by time constraints, leadership support, workplace culture, and organisational infrastructures. OAs showed willingness to participate, yet their prerequisites for engagement were more limited than anticipated. Conclusion: The intervention could be delivered in a real-world home care setting, but delivery, implementation, and activation of proposed mechanisms were constrained by resource limitations, competing priorities, workplace culture, and organisational infrastructures. To improve uptake, intervention adaptations and a stepwise implementation approach are recommended.

## 1. Introduction

Malnutrition (synonym: ‘Undernutrition’ [1]) is a frequent condition among community-dwelling older adults (OAs), in particular those receiving healthcare services, such as those in home care settings [2,3,4,5]. The rate of malnutrition varies from 1.3% to 47.8%, with the highest prevalence in low- and middle-income countries [6]. However, variations in prevalence are partly due to inconsistent assessment methods [4,7,8,9,10].

OAs are at increased risk of malnutrition due to their heightened vulnerability to chronic diseases and polypharmacy, alongside natural age-related changes—including dysphagia, decreased appetite, poor dental status, and reduced taste sensitivity [1,5,11]. Additionally, socioeconomic factors like lower income and living alone further contribute to this increased risk [1,5,11]. The consequences of malnutrition are progressive health decline, diminished functional capacity, greater healthcare utilisation, lower quality of life, early institutionalisation, and higher mortality rates [5,11,12,13].

As the number of OAs worldwide increases, more individuals are expected to face malnutrition [5]; according to the WHO, one in six people globally will be aged 60+ by 2030, with a total number of 1.4 billion people [14]. In Denmark, a minimum of 30% of OAs receiving home care services are malnourished [13], and up to 60% are at risk [15,16,17,18]. In 2024, disease-related malnutrition cost DKK 14.5 billion [13], with DKK 1.4 billion spent on additional home care costs [19].

Although research-based evidence supports nutritional screening as an initial step to address malnutrition [2,20], a significant gap remains between evidence and actual practice in municipal home care settings [4,5,21,22]. While existing research has primarily examined the effectiveness of screening in hospital and primary care settings [23], less attention has been paid to how screening procedures can be embedded in complex real-world contexts. Consequently, little is known about how contextual factors influence the implementation and functioning of malnutrition risk screening interventions in municipal home care settings. As community-dwelling OAs in municipal home care settings frequently present with multifaceted needs related to care, treatment and rehabilitation, nutritional screening should be understood as just one component of a broader, personalised care approach rather than a standalone intervention [2,5].

A scoping review conducted prior to this study identified multiple, interacting barriers contributing to this gap, including insufficient organisational infrastructures, low prioritisation of nutritional care and limited knowledge and awareness among both OAs and staff in home care settings [24]. At the same time, malnutrition among OAs is itself a multifactorial condition, with heterogeneous aetiology and treatment needs that often require tailored, multimodal responses rather than standardised actions [1,5,11].

Together, this clinical and contextual complexity underscores the need for complex interventions that combine screening procedures with implementation strategies and engage interest-holders to enhance acceptability and feasibility of the intervention and thereby improve integration into routine practice [25,26,27,28]. The term ‘interest-holders’ is in this article used to emphasise actors involved in the study with differentiated interests, values, and positions that may shape engagement, processes, and outcomes [29]. Furthermore, early identification of malnutrition risk—rather than detection of established malnutrition—is particularly important among community-dwelling OAs, where timely preventive action may avert further health decline [30].

On this basis, a complex intervention aimed at the early detection of malnutrition risk among community-dwelling OAs (≥65 years) in a Danish municipal home care setting was developed. The intervention comprised an implementation strategy including an interprofessional workgroup and staff learning activities and a screening procedure including an initial start-up conversation and follow-up screenings. It was developed using a hybrid feasibility–implementation approach and underpinned by a context-specific programme theory.

The present study focused on exploring the feasibility and implementation of this intervention in a real-world municipal home care setting. The various frameworks, theories, and methods described in this article were applied to guide this exploration and support the embedded process evaluation.

The development process is reported in a separate article currently under review.

### Aim

The present article describes and presents the findings from a feasibility study of the intervention, including an embedded process evaluation. The aim was to explore the feasibility of the intervention delivery and implementation using quantitative measures of dose, reach, and fidelity, alongside a qualitative exploration of feasibility, acceptability, sustainability, contextual influences, key uncertainties, needed refinements, and mechanism activation, including for whom the intervention’s proposed mechanisms of change were activated. 

## 2. Methods

### 2.1. Design

Overall, the feasibility study employed the MRC/NIHR framework [25]. As the overarching methodological framework, it guided the study design and feasibility evaluation, while other frameworks, theories and methods were applied to support specific aspects of intervention delivery, implementation, process evaluation and exploration of mechanisms. In line with the framework, the development phase was followed by a feasibility phase to assess the acceptability and usability of the intervention and to allow for refinement or adaptation prior to potential scale-up [25,31]. To examine how the intervention functioned, a process evaluation accompanied the feasibility study [32]. In addition, a case study approach was applied to gain insight into for whom the suggested mechanisms of change were activated [33,34,35,36].

The case study methodology [33,34,35,36] allowed an in-depth, multi-level exploration of the dynamic interplay between organisational structures, staff practices, and OA capabilities in the management of malnutrition risk within a real-life setting. Twelve community-dwelling OAs receiving home care services were selected as cases, enabling a multidimensional assessment of the intervention’s feasibility and perceived usefulness by integrating perspectives from OAs, healthcare staff, and, where relevant, informal caregivers. Thereby, each case consisted of an individual older adult and the relevant healthcare staff and informal caregivers involved in that person’s care. By capturing in-depth, multidimensional perspectives, this design facilitated the identification of underlying mechanisms and contextual contingencies that cannot be adequately captured through experimental or survey-based designs alone [35,36]. Further details on case selection are provided in Section 2.5.

To address the gap between evidence-based recommendations and routine practice [4,5,21,22], the integrated Promoting Action on Research Implementation in Health Services (i-PARIHS) framework [37] guided the implementation of the intervention during the feasibility study. The framework supported a continuous focus on facilitation at multiple levels, including different contextual levels, staff, and OAs [37]. Furthermore, the intervention was developed using a hybrid design in which an implementation strategy constituted an integral component of the intervention itself [26,27,28]. This design allowed the simultaneous identification of contextual barriers and facilitators related to both delivery of the screening procedure and the implementation process, thereby enabling iterative improvements to support successful integration into routine practice [27,28].

The feasibility study was conducted in two sequential parts, separated by a participatory workshop, to enable iterative testing and refinement of the intervention in accordance with the MRC framework. Part 1 applied a case study approach with close researcher facilitation and observation to explore feasibility, proposed mechanisms, and contextual influences across 12 older adult cases. Findings from Part 1, together with the participatory workshop, informed refinements to the intervention. Part 2 subsequently examined implementation feasibility within routine home care practice with reduced researcher involvement to explore delivery, uptake, and sustainability under real-world conditions. The feasibility study took place between August 2024 and December 2025. Part 1 took place from October 2024 to August 2025, and Part 2 from September 2025 to December 2025.

### 2.2. Research Questions

Table 1 displays the study’s four research questions (RQs) that the evaluation aimed to address. The four questions structure the presentation of the results.

### 2.3. Target Population and Setting

The target group of the intervention’s screening procedure was community-dwelling OAs, ≥65 years, receiving home care services in a Danish municipality setting. The intervention was designed to be used for all new recipients of home care services. Terminal OAs and OAs previously identified as malnourished or at risk were excluded, as the intervention was designed to identify risk.

All OAs included in the study were recruited from the same single municipal home care unit within the participating municipality.

In Denmark, home care services are provided free of charge to individuals with declining health or functional ability. These services include personal care and practical assistance, such as support with meal preparation and eating, to enable independent living at home. They are primarily delivered by social and health service assistants and helpers, with support from dietitians, nurses, therapists, dementia coordinators, and general practitioners as needed.

Service delivery is governed by a municipal allocation team that determines individual entitlements. Once allocated, services are entered into home care staff’s daily work schedules, referred to as drivers’ lists, which specify the service, recipient, timing, and allotted duration (e.g., 20 min for meal preparation and serving).

### 2.4. The Intervention and Embedded Programme Theory

Embedded in the hybrid design, the intervention consisted of an implementation strategy (Activity A and B) targeting the home care staff and a screening procedure (activity C and D) targeting the OAs, as described in Table 2.

**Figure 1:**
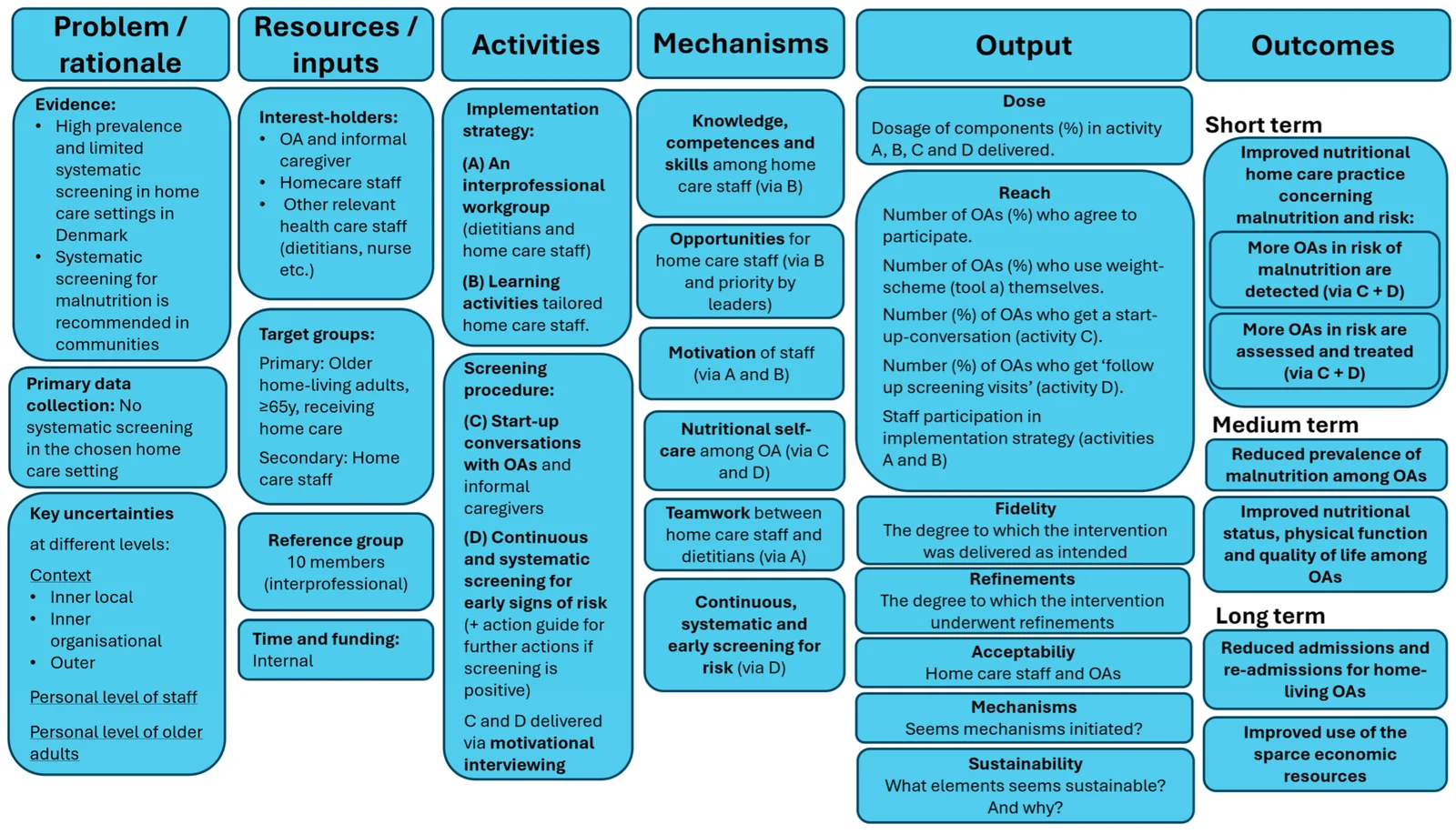
Logic model including mechanisms.

As recommended by Skivington et al. (2021), a programme theory was developed along with the intervention [25]. A programme theory outlines how an intervention is expected to achieve the given outcomes and under which conditions [25]. It includes: (1) key components and their interactions; (2) underlying mechanisms; (3) contextual factors influencing those mechanisms; and (4) reciprocal effects on context [25]. Figure 1 presents a visualisation of the programme theory in the shape of a logic model, created during the development phase (article under review).

The development process was guided by the eleven actions in the IdentifyiNg and assessing different approaches to DEveloping compleX intervention (INDEX) framework [54]. This approach ensured that the intervention and its programme theory were grounded in research-based evidence, selected theories, and primary data collected from the local setting through participatory observations. As previously mentioned, the feasibility study was designed to evaluate the need for refinements and/or adaptations to both the intervention and the programme theory. These needs are presented in Section 3.

In line with Skivington et al. (2021), mechanisms are understood as ‘*the causal link between an exposure (e.g., to some feature of an intervention) and an outcome* [25].’ Accordingly, hypothesised mechanisms are positioned in the logic model (Figure 1) between intervention activities and outputs, while the column of ‘outputs’ guided the evaluation results presented in this article.

The programme theory (mechanisms highlighted in bold) hypothesised that, supported by leadership prioritisation (i.e., organisational **opportunity**), the interprofessional workgroup (Activity A) and tailored learning activities (Activity B) would enhance collaboration between dietitians and home care staff and strengthen staff **motivation** and **capability** to deliver the screening procedure (Activities C and D). Through delivery of the screening procedure, it was further hypothesised that OAs would gain improved **nutritional self-care**. Collectively, delivery of all activities was expected to contribute to **continuous, systematic, and early detection of malnutrition risk**, leading to subsequent assessment and treatment and, ultimately, to a reduction in malnutrition prevalence.

Selected theories were used to substantiate the choice of activities and their corresponding mechanisms of change within the programme theory [25].

For home care staff, the COM-B theory of behaviour change [41] informed the hypothesis that the intervention activities would support staff capability, opportunity and motivation to perform the screening procedure, thereby facilitating behavioural change in practice.

For OAs, activities and corresponding mechanisms of change were informed by findings from a scoping review conducted as part of the development phase. This review indicated that limited knowledge of and awareness of malnutrition and nutritional recommendations constitute important barriers to OAs’ engagement in nutritional interventions [24]. The review further highlighted that self-care and the maintenance of independence are highly valued by OAs, while tailored support that respects autonomy is perceived as a key facilitator [24]. Based on these findings, and drawing on the theory of self-care by Riegel et al. [46] and principles of motivational interviewing [47], the programme theory hypothesised that motivational interviewing combined with increased individually tailored knowledge and awareness would engage OAs in the intervention. This engagement was expected to support their nutritional self-care—e.g., to monitor their own body weight—and thus contribute to early identification and prevention of malnutrition.

### 2.5. Interest-Holders Engaged in the Feasibility Study

Interest-holders’ perspectives were consistently explored throughout the test period to assess acceptability and feasibility. Relevant key stakeholder groups were identified during the development phase (article under review). They are presented in Table 3, including our rationale for involvement.

A total of 28 OAs were identified for participation: 12 in Part 1 and 16 in Part 2. The 12 OAs who participated as cases in the first part were chosen via strategic selection, with maximum variation to provide as much information as possible about the phenomenon and clarify the deeper causes of behaviour in relation to the intervention [55]. Despite potential dropouts, 12 strategically selected cases were considered adequate for identifying key uncertainties and constraints and allowing for both in-depth analysis and variation [56].

In Part 2, the focus was mainly on the organisational level and staff practice. Therefore, each member of the home care staff involved was asked to choose one OA, leading to the identification of 16 eligible OAs.

**Table 3:** Interest-holders’ involvement in the development process.

Interest-Holder Group	Number of Participants and Characteristics (Pseudonymised Identifiers *)	Rationale for Involvement	Engagement in the Study Process
(1) OAs	28 OAs were identified as eligible for participation (B3–B31), 12 in Part 1 and 16 in Part 2. Part 1: 12 accepted. Age: 72–98 years; duration: 8–398 days. Part 2: 5 ‘new’ accepted (2 declined; 9 did not receive any visits.); plus 7 ‘repeaters’ from Part 1. Age (‘new’): 63 **–96 years; duration (‘new’): 70–77 days.	They are the receivers of the screening procedure in the intervention.	Development phase Part 1: Case study, interview 1. Part 2: Interview 2.
(2) Home care staff	4 helpers and 3 assistants (P1, P3, P10, P13, P16, P17, P18)	They are the primary staff group in the home care setting, maintaining daily contact with OAs. Additionally, they play a central and coordinating role between OAs and other staff groups.	Development phase WS Focus group 1 Reference group and key group
(3) Dietitians	3 clinical dietitians (D1, D2, D3)	They are the nutritional experts in the municipality setting.	Development phase WS Focus group 2 Reference group
(4) Leadership	5 managers: daily home care manager, area manager for home care, area manager for dietitians, senior and health manager, area manager for nurses (L1, L3, L4, L5, L6)	They are the decision-makers. The prioritisation by managers is mandatory if new interventions are to be implemented and sustain in practice [37].	Development phase WS Focus group 3 Gatekeeper Reference group
(5) Nurse	1 home care nurse (S2)	They participate in weekly home care meetings and help home care staff coordinate with other staff groups.	Development phase WS
(6) Allocation Team	3 members: head of allocation team, case manager and care coordinator (V2, V3, V4)	All home care services are allocated by a case manager. In Part 1, the allocation process was identified as a main barrier for feasibility, indicating the importance of their involvement.	WS
(7) Occupational therapist	1 occupational therapist (E1) (E1 is employed as part of the home care group)	They are responsible for the rehabilitation programmes offered to the OAs. Nutrition should be a part of such programmes.	WS Reference group and key group
(8) Dementia coordinator	1 dementia coordinator	Cognitive impairments and dementia are prevalent among the OAs, and malnutrition is prevalent among OAs with dementia [57]. In Part 2, OAs with cognitive impairments were included.	WS: Intended to participate but was absent.
Reference group	P3, P10, P13, D1, D2, D3, L1	Group members acted as ‘intern facilitators’ to enhance implementation [37].	(see above)
“Key group”	P3, P10, P13, E1	Group members acted as ‘champions’ and mentors for colleagues in Part 2.	(see above)
Research team	TL, HK and PL.	The researchers.	Designed the research and maintained data collection and analysis.

Abbreviations: WS = participatory workshop. * Participants were pseudonymised using alphanumeric codes (e.g., B3–B31) to protect confidentiality. ** B16 and B17 were <65 y, indicating that home care staff chose outside ‘Target-group-criteria’. However, B16 and B17 did not get any visits.

### 2.6. Ethical Approval

Prior to study initiation, the project was assessed by relevant Danish research ethics authorities and deemed exempt from notification requirements. Further details are provided in the Institutional Review Board Statement. 

Although formal ethical approval was not required under Danish legislation, the study was conducted in accordance with international ethical guidelines for research involving human participants, including the Declaration of Helsinki [58]. Informed written or oral consent was obtained from all participants to ensure voluntary participation and transparency regarding data use.

The study further adhered to the Danish Code of Conduct for Research Integrity [59]. The researcher’s institution acted as data controller, and all data were stored securely in compliance with the GDPR and will be deleted when no longer required, or no later than 30 June 2031.

### 2.7. Data Collection and Analysis

Data collection methods:

The triangulated data collection strategy was chosen to maximise credibility by capturing complementary perspectives on mechanism activation, thereby enabling the investigation of the RQs and the measurement of outputs as demonstrated in the logic model.

Participant observations and informal interviews [60,61] were conducted by TL throughout the feasibility study during visits to OAs, weekly home care meetings, learning sessions, and discussions with managers, the reference group, or staff members. While observations provided insight into current practices and interest-holder engagement with the intervention, informal interviews helped deepen the understanding of the observed and provided insights into decision-making processes and interest-holders’ experiences with the intervention [62,63]. Field notes were taken during observations and interviews to ensure the details were remembered [64].

NEXUS (Systematic A/S, Aarhus, Denmark), the electronic patient record, provided objective data on delivery to assess ‘*dose*’, ‘*reach*’, and ‘*fidelity*’ across the intervention activities. Dose referred to the amount of intervention delivered (e.g., meetings conducted, learning activities delivered, start-up conversations completed, and follow-up screenings conducted). Reach referred to participation and uptake among the intended target groups (e.g., attendance by staff members and participation of OAs). Fidelity referred to the extent to which intervention activities were delivered according to protocol, based on available documentation in NEXUS. However, as not all delivered components may have been documented, qualitative data supplemented the register data, which allowed for triangulation and a more nuanced understanding of actual intervention delivery.

Semi-structured individual interviews [65] were conducted with OAs in both Part 1 and Part 2 of the feasibility study to explore how they interacted with the intervention, how it affected them, and which factors influenced their prerequisites for nutritional self-care as hypothesised in the programme theory. In Part 1, face-to-face interviews lasting 40–82 min were conducted, while shorter follow-up telephone interviews lasting between 1 and 20 min were conducted in Part 2 to reduce participant burden and facilitate brief reflective feedback in a less formal setting. Three OAs participated in interviews in both parts. All interviews were conducted by TL using a semi-structured interview guide informed by the EQ-5D-5L instrument [66] and Riegel et al.’s self-care theory [46] to support systematic exploration of OAs’ prerequisites for engagement in the intervention and investigate whether the hypothesised mechanisms were activated. All interviews were electronically recorded and transcribed by TL shortly afterwards.

A participatory workshop using the World Café method [67,68,69,70] was conducted to actively engage interest-holders in addressing uncertainties identified in Part 1 and to make agreed refinements prior to Part 2. Participants progressed through three rounds of discussion, during which they contributed perspectives, identified challenges, and suggested refinements. TL opened and closed the workshop. The identified uncertainties and the planned refinements and adaptations are presented in Section 3. The discussions were electronically recorded and transcribed by TL shortly afterwards.

Focus group interviews with three personnel groups were conducted in the last part of the feasibility study to explore the participants’ experiences, perceptions, and attitudes toward the intervention, and to investigate the social dynamics of their discussions [71,72]. The durations of the focus groups were as follows: focus group 1: home care staff (90 min), focus group 2: dietitians (60 min), and focus group 3: managers (60 min). The sequence was informed by the BIKVA model [73], allowing findings from staff and dietitians to inform the leader interviews. All focus groups were moderated by TL, with PL or HK acting as observers to support the moderation and strengthen analytical rigour [71,72]. A semi-structured interview guide, informed by empirically identified uncertainties and selected frameworks including situated learning theory [39], i-PARIHS [37], and MRC process evaluation [25] was used to explore whether hypothesised mechanisms were activated and contextual influences. All interviews were electronically recorded and transcribed by TL shortly afterwards.

**Figure 2:**
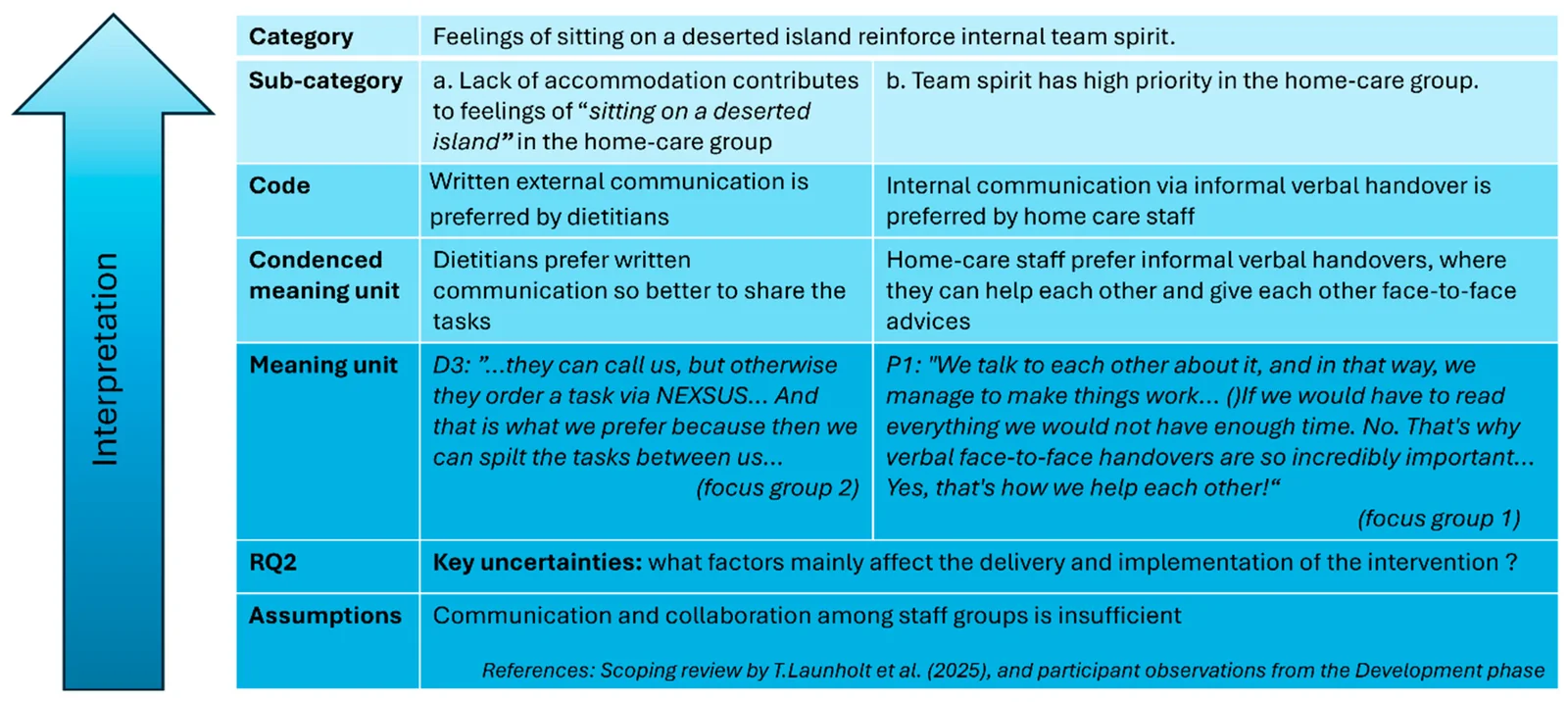
Examples of consistency between RQ, meaning units, condensed meaning units, codes and category for RQ 2. Assumptions were based on a Scoping review [24] and participant observations from the development phase.

Analysis:

An inductive qualitative content analysis was employed to enable an open exploration of the qualitative data, with categories generated directly from the material [74,75,76,77]. In line with established methodological guidance, the RQs and suggestions in the programme theory were used to guide analytic focus and relevance without predefining coding structures, thereby allowing categories to emerge inductively while maintaining coherence between data, analysis and study aims [74,75,76,77]. Data management was supported using NVivo 14 (Lumivero, Denver, CO, USA) [78]. The analysis was led by TL and reviewed with HK and PL to strengthen analytical rigour and intercoder reliability [74,76,77]. Recognising that qualitative data are co-constructed through interaction, the researcher maintained reflexive awareness of their influence throughout data collection and analysis, addressing this through triangulation of data sources and collaborative analytic review [75]. Figure 2 illustrates an example of the analytic steps.

NEXUS data were analysed using descriptive statistics including frequencies, proportions, and percentages [79]. These analyses were used to assess dose, reach, and fidelity for each intervention activity as part of the process evaluation. Where NEXUS documentation was insufficient to assess fidelity, qualitative data were used to supplement and contextualise the quantitative findings.

## 3. Results

Findings are synthesised in this section to explore outputs shown in the logic model and to answer the RQs one by one. The findings were generated across two sequential parts of the feasibility study. Part 1 involved an in-depth case study exploration with close researcher facilitation and observation to explore feasibility, perceived usefulness, mechanisms and contextual influences across 12 OA cases. Findings from Part 1, together with the participatory workshop, informed refinements to the intervention. Part 2 focused on implementation feasibility, exploring delivery uptake, and sustainability under routine home care conditions. The final section presents refinements and adaptations made between Parts 1 and 2 of the study.

### 3.1. Delivery of the Intervention

To investigate to what extent the intervention activities—described in Table 2—were delivered as planned, ‘dose’, ‘reach’, and ‘fidelity’ [32,80,81] were evaluated individually for each activity, as shown in Table 4, Table 5, Table 6 and Table 7.

#### Dose, Reach, and Fidelity

Activity A:

The interprofessional workgroup held eight meetings instead of the thirteen originally planned, as leadership opted to limit the use of staff resources. According to the protocol, the workgroup was intended to function as a ‘reference group’. This fostered active participation from members in meetings to identify key uncertainties and propose refinements to the intervention. However, although TL encouraged active participation among group members, she remained in control throughout the project period. Members attended meetings when they were scheduled in their calendars, reflecting routine organisational practice. Participation thus primarily constituted presence within an established governance structure rather than shared decision-making.

Activity B:

All planned e-learning and dietitian-led sessions were delivered (I and II). Reach was reduced due to staff absence related to annual leave, illness or competing priorities. Use of mentor assistance (III) declined markedly in Part 2, with only three of 16 staff members accepting assistance.

Activity C:

In Part 1, TL mentored all start-up conversations in the screening procedure as planned. In Part 2, home care staff members conducted five out of 16 planned conversations, and qualitative data suggest that only selected components were delivered, reflecting a low dose of delivery. According to protocol, a trained professional—e.g., a dietitian—should have led the conversations to improve the possibility of motivational interviewing [47]. However, due to managerial reluctance, this was not possible. See ‘Refinements and adaptations’ for further details.

Activity D:

TL initially acted as a mentor for home care staff in the key group in Part 1, and subsequently, when documentation in NEXUS indicated a need or upon request from staff. TL did not provide mentoring to ‘new’ staff who commenced in Part 2.

During the test period, it seemed clear that TL’s involvement and allocation of time, plus specific notes on ‘the drivers’ lists’ regarding the screening procedure, had a major effect on delivery. Home care staff rely heavily on these lists to structure daily work, making explicit scheduling a prerequisite for delivery. Consequently, TL was required to ensure continued inclusion of the screening procedure on the ‘drivers’ lists’ throughout the test period by securing managerial support for resource allocation and by repeatedly reminding them to include these notes.

In Part 2, refinements were introduced to improve scheduling procedures. However, as shown in Table 7, these refinements did not appear to have been successfully introduced.

In summary: Delivery patterns illustrated partial but inconsistent feasibility. High delivery in Part 1 depended heavily on facilitation by TL. Declines in Part 2 suggest insufficient integration into routine practice.

### 3.2. Key Uncertainties: Factors That Affected the Delivery and Implementation of the Intervention

Several key uncertainties were identified, reflecting misalignment between programme theory assumptions and real-world capacity. These related to current practice, the intervention itself, and interest-holder engagement.

#### 3.2.1. Current Practice

Home care staff described their job function as multifaceted. They coordinate teamwork among health professionals and play vital roles for OAs. As they put it:

P1: ‘*We wear many hats when we’re at work.’* P16*: ‘Yes, we’re the eyes and ears’* (focus group 1). However, they experience limited decision-making authority and slow communication with other staff groups, contributing to a sense of isolation.

P16: ‘*And it’s often the nurse who has more authority and can make things happen… because, you know…we simply don’t have the competence to do so.*’ (focus group 1). L1: ‘*…at times you feel like you’re sitting on a desert island*’ (meeting with manager).

These feelings of isolation strengthen their internal team spirit, leading them to value informal, non-scheduled face-to-face peer support. As P1 explains, ‘*We talk to each other about it, and by doing so, we manage to make things work*… () … *That’s why verbal face-to-face handovers are so incredibly important… Yes, that’s how we help each other!’* (focus group 1).

Preventive nutritional care is not systematically prioritised in current practice and often preventive work is displaced by urgent tasks, described as ‘*firefighting*’ (D3 in focus group 2). Furthermore, allocation of time for malnutrition risk screening is met with significant resistance from the allocation team. As mentioned by L1: ‘*Yes, the Allocation Team should be nudged because… We’re always too late… we don’t act until the clothes are hanging off them…*’ (session 2 with dietitians).

#### 3.2.2. The Intervention

Strengthened practice despite challenges in acceptance

Members of the key group acted as *‘champions’* in the feasibility study. They valued the screening scheme and weight measurements and described the intervention as an ‘*eye-opener*’ (P16) that legitimises preventive work and supports more standardised practices:

P3: ‘*It’s a focus that’s been missing.*’ P18: ‘… *previously, it used to be a real struggle with the Allocation Team. Discussions about whether it (=weight monitoring) was really necessary*.’ P3: ‘…*and so that everyone does it in more or less the same way*’.

(focus group 1)

During Part 2, these positive experiences were actively used as the champions engaged colleagues through learning sessions. However, their efforts were challenged and met with resistance, as limited collective acceptance was observed among some of the champions’ colleagues in the wider staff group.

Authority of the handbook.

The ‘handbook’ is a guidebook on best practices, and it serves as a formal guideline for staff, including instructions based on the Danish Health Authority’s recommendations [15], such as recommending monthly weight measurements of OAs to screen for risk of malnutrition. The intervention draws on the same underlying evidence. However, despite the intended role of the ‘handbook’, staff appeared to be only vaguely familiar with the handbook and rarely used it:

D1: ‘*Maybe there’s just one person who actually uses it*’.

(focus group 2)

Furthermore, managers seem unclear about its authority, and its practical implementation appears constrained by more immediate ‘*firefighting*’ priorities, making it difficult for managers to allocate the necessary resources.

This directly challenges intervention delivery, suggesting that the intervention risks being treated similarly and not integrated into daily practice [37].

#### 3.2.3. Interest-Holder Engagement

From the outset, the engagement of interest-holders was a core element [25]. However, active engagement was challenged across staff groups and OAs.

Home care staff generally seemed used to directive ways of working, and some were hesitant when invited to take an active role in refining the intervention. However, engagement varied across the group, and towards the end of the study, some champions showed ownership by leading learning sessions and actively involving colleagues in the intervention.

Dietitians expressed reluctance based on previous experiences and structural constraints:

D1: ‘*We cannot change the framework… I’ve been trying for 12 years. We have to do the best we can within the framework*’.

(Session 3, led by dietitians)

D1: ‘*My concern is that this intervention will end up ‘dying’ like the previous one. How can we make sure it doesn’t happen this time?*’.

(focus group 2)

Interviews with OAs indicated that they perceived preventive work, including the intervention, as unnecessary, because they considered themselves to be at low risk:

*B4: ‘Yes, but I don’t really have any issues with any of it’*.

(phone interview with B4)

However, OAs agreed to participate in the intervention and did not object to receiving the screening visits, including weighing.

### 3.3. Mechanisms: Activation of Intended Mechanisms

This section examines whether the mechanisms hypothesised in the programme theory were activated as intended and how contextual factors influenced their activation. Mechanisms related to home care staff and OAs are presented separately.

#### 3.3.1. Home Care Staff: Capability, Motivation and Opportunity

In line with the programme theory and the COM-B model [41], motivation, capability, and opportunity were prerequisites for home care staff to deliver the screening procedure as intended. Findings showed partial delivery of the screening procedure (RQ1, Table 6 and Table 7), indicating some gains in staff capability through the learning activities. However, delivery was highly dependent on TL’s facilitation of priorities, time allocation, and scheduling, illustrating how contextual constraints limited opportunity for mechanism activation.

In Part 2, delivery declined further as TL adopted a more withdrawn role to test the feasibility and actual uptake in practice. In this part, staff were required to actively request mentor assistance, which only three of sixteen did. The reasons for this remain unclear. It may be due to low awareness or low motivation. As described previously, collective motivation appeared limited and could be a contributing factor.

Despite challenges with motivation and opportunity, the learning activities (Activity B) did support capability development. Staff valued peer-based and situated learning; they perceived the screening as simple and requiring limited training:

P16: ‘*I couldn’t quite remember it… so I called P3, was guided, and figured it out*’.

(focus group 1)

P3: ‘*And it’s not something you need to be trained on 50 times*’.

(focus group 1)

This was supported by leadership perspectives, emphasising the importance for staff having ‘*something concrete to discuss*’ (L1 at a leader meeting). Observations during TL’s mentoring of the key group indicated improved fidelity over time, suggesting gradual capability development, albeit with notable individual differences among staff in learning capabilities.

Interprofessional workgroup meetings (Activity A), combined with dietitian-led sessions and e-learning (Activity B), aimed to strengthen collaboration and clarify roles between home care staff and dietitians. The increased physical presence of the dietitians through weekly meetings initiated during the project period, together with dietitian-led sessions and e-learning sessions, appeared to have enhanced teamwork among the two staff groups and, consequently, the competencies of home care staff. As highlighted by the dietitians: D3: ‘*… it* (=physical presence) *gives more enquiries, and staff are more likely to call us.*’ D2: ‘*they remember us and reach out to us…*’ (focus group 2).

To sum up: Staff capability increased through situated learning, as anticipated in the programme theory. However, the mechanisms were only partially activated, as motivation and opportunity remained structurally constrained.

#### 3.3.2. OAs: Nutritional Self-Care

According to the programme theory, delivery of the screening procedure was expected to enhance OAs’ nutritional self-care. However, this mechanism was largely not activated. Early in the test period, OAs showed limited engagement. Interview and observational data suggested that this was primarily due to limited prerequisites and motivation, rather than delivery quality alone.

Declining health, including chronic illness, pain, fatigue and a fear of falling, constrained both physical and cognitive resources:

B4: ‘*I have neither the energy nor the strength for anything anymore.*’

B5: ‘*I sleep a lot. I am simply so tired.*’

Age and life situation further influenced motivation, particularly the desire for self-determination and perceived lack of meaning in behaviour change:

B3: ‘*I’m almost 100 years old… now I shall decide for myself.*’

Prerequisites for self-care were also shaped by limited access to research-based knowledge. Instead, decision-making was grounded in lifelong habits, cultural norms, taste preferences, economic considerations, social networks, and guidance from authority figures, as illustrated by multiple interviews:

Cultural norms: TL: *“…why do you want a longer walk?”* B3: *“Well, I have always enjoyed being out in the fresh air… I have been a farmer’s wife all my life, and we certainly weren’t allowed to just sit inside and look pretty”* (she laughs).

Economic considerations: B6: *“…Sometimes there are two slices of meat, and then I save one for my evening meal”.*

Social network: B11: “*Well, food doesn’t mean anything to me… Only on Sundays when we sit and enjoy ourselves”* (their daughter visits every Sunday).

Authority: TL: “*So, who says that?”* B4: “*Yes, actually. I can’t remember… the municipality or the doctor… I think it was the doctor.”*

Behavioural change based on increased knowledge and awareness, as assumed in the programme theory, was therefore challenged. However, responsiveness to authority figures appeared to support acceptance of participation.

To sum up: Self-care mechanisms suggested by the programme theory primarily failed to activate. This pattern suggests that self-care mechanisms are sensitive both to delivery conditions and OAs’ individual prerequisites.

### 3.4. Sustainability: Part of the Intervention That Will Be Integrated into the Ongoing Daily Practice

Focus group interviews with staff and leaders indicate that some intervention elements are likely to sustain or seem planned for future sustainability, reflecting successful facilitation. Guided by the i-PARIHS framework [37], facilitation of the intervention’s implementation was carried out consistently throughout the feasibility study, with attention given to various contextual levels besides OAs and staff preferences, which contributed to its sustainability. Sustainability in the study was linked to the reactivation of existing practice ideals—such as the intention to follow the handbook—which were not consistently enacted prior to the intervention. In this way, the intervention contributed to translating these ideals into practice by supporting competence development and enabling staff and managers to identify feasible solutions aligned with available resources, staff capacity, and organisational infrastructures.

Additionally, sustainability for OAs was enhanced by tailoring the intervention to fit their capacity and resources, along with addressing their habits of authoritative guidance.

Sustainable elements—together with actions or initiatives that supported their sustainability—are listed below, including selected quotations for demonstration purposes.

#### 3.4.1. Weight Monitoring of OAs

Continuous preventive weight monitoring of OAs is perceived as a future standard practice, contingent on prioritisation and time allocation:

L6: ‘*If we want to prioritise it, we need to do so… No OAs shall be left behind!*’.

(focus group 3)

P16: ‘*I think that all OAs will have their weight monitored regularly. It will become part of our home care practice*’.

(focus group 1)

Sustainability of this element was strengthened by renewed leadership commitment to preventive work, changes in resource allocation following the new ‘Senior Citizens Act’, and OAs’ acceptance of staff-led weight monitoring. However, routine monitoring was not fully embedded during the project and should be considered an indicator of potential rather than achieved sustainability.

#### 3.4.2. Dietitians’ Physical Presence

Dietitians plan to rotate visits across home care districts as a resource-efficient strategy to support collaboration with home care staff:

PL: ‘*So physical presence matters?’* D3: *‘Yes, it does… it gives more enquiries, and staff are more likely to call us*’.

(focus group 2)

D2: ‘*They remember us and reach out to us*’.

(focus group 2)

Furthermore, leaders expressed intentions to maintain this model, indicating organisational support for its sustainability.

#### 3.4.3. Start-Up Conversation

The start-up conversation for new home care recipients is recognised as valuable, and a tailored version is under development:

L1: ‘*Prompted by this project, we are in the process of creating material for a start-up conversation… explaining why weight measurements are important*’.

(focus group 3)

#### 3.4.4. Systematic Screening

Systematic screening, as anticipated, will not be part of the current practice at present. However, partial integration of some part of the screening tool scheme (Activity D) into existing infrastructure is considered feasible:

L1: ‘*Maybe we could use it* (=screening tool scheme) *to supply our Triage scheme that we already use. We could include three sub-questions about nutrition, couldn’t we? So, we selected the most essential ones’*.

(focus group 3)

### 3.5. Summary of Barriers Identified Across the Feasibility Study

The findings presented across the four research questions identified several interrelated barriers that influenced intervention delivery, implementation, activation of proposed mechanisms, and sustainability. To provide an overview of these findings, the barriers are summarised in Table 8.

### 3.6. Refinements and Adaptations

To improve feasibility, refinements were made between Parts 1 and 2 of the feasibility study [25]. According to Skivington et al. (2021), refinement is the process of ‘fine tuning’ the intervention, without making significant changes to the programme theory [25]. Refinements made are listed below; whether these may have changed features of the programme theory will be considered in Section 4.

Motivational interviewing [47], as anticipated in the programme theory, was deprioritised due to limited staff capacity and managerial priorities.

Start-up conversations and the screening tool were simplified to fit time constraints. Height, BMI, and calf circumference measurements were excluded from the start-up conversations, and the layout of the screening tool scheme was streamlined to fit on one page instead of three.

Allocation of time and explicit notes on ‘drivers’ lists’ were agreed with relevant managers and the allocation team to ensure the intervention was tested, as preventive work in current practice is often set aside due to more urgent issues.

Integration of the screening tool scheme into NEXUS was explored, as it would ease home care staff’s opportunity to use the tool, given that ‘paper versions’ reduced feasibility. However, integration proved technically impossible.

Automation. Home care staff are in front, but they have no specialised and updated nutrition knowledge, and they prefer clear, direct communication. To ease their workload, the day-to-day manager therefore requested that positive screening should automatically trigger the involvement of other staff groups, for example, through discussion at the weekly interdisciplinary triage meeting.

OAs with cognitive impairments represent a significant group in the home care setting, and they are at high risk [82]. They should therefore be included. As it was ethically acceptable to include them, we decided to do so in Part 2.

## 4. Discussion

This article aimed to outline findings from a feasibility study, including process evaluation, of a newly developed intervention aimed at early detection of malnutrition risk among community-dwelling OAs (≥65 years) in a Danish home care setting.

Overall, the intervention was found acceptable and feasible to staff and OAs. However, delivery and implementation were consistently affected by several contextual factors, including time constraints, leadership support, culture and fit with existing organisational infrastructures. These findings highlight a recurrent challenge in complex intervention research, namely that theoretically grounded programme theories often require adjustment when confronted with real-world practice conditions. In this regard, the feasibility phase functioned not only as a test of intervention components, but also as a critical empirical examination of assumptions embedded in the initial programme theory.

From a theory-practice perspective, these findings highlight how implementation in practice can necessitate adaptation of theoretically grounded assumptions. While the programme theory was primarily developed prior to implementation and involved interest-holders as well as field observations, the process evaluation revealed areas where closer involvement of practice actors earlier in the development phase and more extensive use of field observations might have enabled better anticipation of contextual constraints. At the same time, the present design enabled systematic empirical testing of a theory-informed intervention, thereby providing a transparent basis for refining the programme theory in light of real-world conditions rather than speculative adaptation.

Highlights from the findings are discussed below, alongside implications for future research aiming to bridge the gap between research-based recommendations and routine home care practice.

### 4.1. Highlights with Implications for Practice and Future Research

#### 4.1.1. Clarity in Situated Knowledge

*‘You won’t act if you don’t know what to act on.*’ This statement, made by a home care manager during the development phase, highlights the need for home care staff to possess relevant knowledge and skills. We based the intervention’s learning activities on situated learning, which enabled staff to develop competencies within their community of practice [39,83,84]. Field observations confirmed that this approach suited home care staff with a strong team culture [85,86]. However, the evaluation also indicated that further simplification and clarification were needed—ideally through automatised follow-up actions when screening is positive—to reduce workload and decision burden. This aligns with Højrup’s wage-earner lifestyle theory, which suggests that work under high time pressure, physical workload and limited autonomy favours clear, efficient and standardised procedures that reduce uncertainty and cognitive demands [45]. From this perspective, ‘automation’ is not merely a productivity measure, but a culturally congruent response to wage-earner-organised work practices where predictable routines protect workflow under strain. Although automation was agreed upon during the project as a desirable refinement, it was not sufficiently tested in practice. Future research should therefore develop and evaluate automated follow-up processes to reduce workload and improve fit with everyday work culture.

#### 4.1.2. Isolation as a Barrier to Change

During the test period, we observed how home care staff occupied key coordinating roles in which they were responsible for bringing observations and concerns forward to other staff groups. These roles contributed to the development of an internal team spirit and a shared sense of responsibility. However, at the same time, these roles generated a sense of isolation, reinforced by their responsibility without decision-making authority and insufficient communication with other staff groups. These findings were mirrored in a study by Bergqvist et al. (2024), who similarly report that home care staff often feel proud but powerless, operating with high responsibility but limited authority, insufficient organisational support, and weak integration with other healthcare actors [87]. The sense of isolation is further intensified by the burdensome communication processes characteristic of home care work, a challenge also documented in the study by Hald et al. (2024), who show that communication conditions shape staff performance and that role structures, responsibilities, and task load influence collaboration, which supports the interpretation that communication barriers undermine teamwork and reinforce isolation [88].

In our setting, home care staff furthermore managed numerous heterogeneous tasks among OAs, with limited structural opportunities for ongoing competence development to ensure tasks matched their competencies. This is also consistent with findings from Bergqvist et al. (2024) [87].

These structural conditions make it unsurprising that home care staff might display resistance toward new initiatives—such as the intervention—as we saw in Part 2 of the study. The staff group thrives under clear procedures and instructions, which new external initiatives can disrupt [45]. Furthermore, the sense of isolation, limited authority, and unstable interdisciplinary support amplify this resistance. The absence of a well-functioning interdisciplinary organisation appears particularly influential, as emphasised in studies by Hald et al. (2024) and Slåtsveen et al. (2024) [88,89]. The two studies highlight how insufficient collaboration with other professional groups reinforces home care staff’s isolation and weakens their capability to support change processes [88,89]. In contrast, enhanced interdisciplinary collaboration—such as through interdisciplinary teams—will strengthen communication, create shared responsibility, and reduce the sense of having to make complex decisions alone [88,89]. This is consistent with the VIVE report (2021), which concludes that interdisciplinary team structures provide a stronger foundation for cooperation and reduce isolation among frontline staff [86].

Although cultural behavioural changes can be challenging to achieve [37,90], our findings suggest that making such changes through reformed organisational infrastructures will reduce feelings of isolation, encourage interdisciplinary collaboration, and decrease resistance to new innovations. The new ‘Senior Citizens Act’ recommends multidisciplinary teams in home care settings, which could provide a pathway for these proposed improvements.

#### 4.1.3. The Role of the Dietitians

Originally, our intention was for dietitians to take on a more active role by conducting start-up conversations alongside home care staff and applying motivational interviewing. However, resource limitations made us modify the intervention and assign dietitians a more supportive role. They therefore only attended meetings and led selected sessions. This modification is consistent with our scoping review from 2025 [24], which showed that dietitians frequently facilitate learning sessions for other staff groups to optimise use of their scarce resources [24]. Moreover, this is supported by ESPEN’s blue book, which highlights that ‘non-specialist caregivers’ can perform the screening function [91]. However, ESPEN emphasises further that professional clinical judgement is essential to correctly assess malnutrition [91]. In situated learning environments, procedural actions may be encouraged over reflective ones, as we observed. As stressed by ESPEN, it is therefore crucial that home care staff are met with appropriate support for subsequent actions [91]. Our refinements in Part 2 reflected this need, and from a situated learning perspective, automatically triggering involvement from other professional groups after a positive screening can be understood as a structural support that sustains collective practice and aligns tasks with professional competencies.

#### 4.1.4. “Welcome to Home Care—We Need to Measure Your Weight”

One home care staff member offered this remark, laughing afterwards. The statement illustrated that practices touching on intimate or socially sensitive domains require cultural normalisation before they can be sustainably integrated into everyday care. Weight measurement is often perceived by both OAs and staff as private or taboo, positioning it at the boundary of what is considered legitimate home care work [92]. Such boundary-work highlights how professional identities and norms shape what is experienced as appropriate practice within a community of care [40]. From a situated-learning perspective, routinisation therefore becomes essential—because only when a sensitive activity is embedded as a standardised element of the care trajectory does it shift from an intrusive request to an accepted component of the shared repertoire of the community of practice [40]. The emerging notion of a ‘start-up conversation’ reflects the need for structured entry points that legitimise a new practice of weight monitoring and support its gradual incorporation into routine home care. Integrating such ‘start-up conversations’ into standard procedures—together with clear information about the purpose of weight monitoring—may therefore facilitate normalisation of ongoing monitoring and strengthen the implementation of malnutrition risk screening.

#### 4.1.5. Constraints on Nutritional Agency in Later Life:

Overall, the findings indicate that the nutritional habits of OAs were predominantly shaped by routine, social influences, and cultural food practices rather than by conscious or reflective decision-making. Limited consideration of alternative food options can therefore be understood as part of a broader pattern in which everyday food choices are largely habitual and pragmatically oriented. From a capacity-oriented perspective, autonomy in nutritional self-care depends on the interplay between motivation and capacity, where functional decline and multimorbidity may constrain the translation of intentions into action [93]. This interpretation aligns with theories of ageing that conceptualise later life as a dynamic movement along a continuum from independence to dependence, shaped by interactions within social networks and healthcare systems [94]. Behavioural research similarly suggests that knowledge alone rarely leads to behavioural change, particularly in preventive domains in which habits are deeply embedded and typically disrupted only in response to illness or acute health events, as also reflected in recent findings from the Dietary Habits of the Danish People report (2026) [95]. These insights are consistent with the self-care theory proposed by Riegel et al. (2012) [46], which informed the programme theory and supported the inclusion of motivational interviewing. However, the feasibility findings indicate that limited capacity among OAs, combined with organisational constraints within municipal home care, restricted both the feasibility and the practical relevance of motivational strategies. Consequently, the programme theory was adapted during the test period to better accommodate the capacities of older adults and the available staff resources. Further adjustments should be evaluated prior to subsequent research.

#### 4.1.6. Implications for the Implementation of Preventive Interventions

Guided by the i-PARIHS framework [37], this study underscored how the implementation of preventive interventions in home care depends on alignment between facilitation, leadership, and organisational and policy context. Consistent with the COM-B model informing the programme theory [41], home care staff required not only capability (skills and knowledge) but also opportunity, defined as the organisational conditions that enable preventive practices to be prioritised and enacted, including leadership support and protected time.

Across the evaluation period, findings showed how these organisational conditions repeatedly limited opportunities for initiating intended programme mechanisms. Despite facilitation strategies aligned with i-PARIHS, such as tailoring intervention components, reducing time demands, and engaging managers, preventive activities struggled to compete with more acute and operationally urgent tasks.

Changes in the outer context further emphasised this point. The introduction of the new ‘Senior Citizens Act’ during the latter part of the test period altered financial and managerial autonomy, demonstrated how policy-level shifts can both create opportunities and introduced uncertainty regarding the prioritisation of prevention. From an i-PARIHS perspective, this underscores that sustainable implementation requires coherence across system levels, including organisational infrastructure and political commitment, rather than reliance on local adaptation alone [37].

Our findings reflect the well-documented knowledge–practice gap, where evidence-based recommendations are inconsistently implemented or sustained [81,96,97]. Furthermore, systematic review evidence highlights that barriers such as limited awareness, competing priorities, and insufficient organisational integration commonly hinder guideline uptake [98], while limited translation into practice has been linked to insufficient specification of mechanisms of change [99]. Although mechanisms of change were explicitly articulated in this study, their activation was partly constrained by contextual factors, such as leadership hesitancy to allocate resources.

Moreover, our findings indicate that preventive interventions are particularly vulnerable in practice, as they compete with immediate care demands rather than being structurally prioritised. This underscores a broader implementation challenge: facilitation alone is insufficient without embedding preventive practices within existing resourcing and prioritisation systems [81,100].

Taken together, our findings are consistent with prior research across healthcare contexts but add context-specific insight into how these well-known implementation challenges unfold in home care. They suggest that, for preventive interventions to be successfully implemented and sustained, they must be supported by explicit organisational and policy-level resourcing mechanisms. Without such alignment, even well-designed and acceptable interventions risk remaining vulnerable to competing demands, limiting their integration into routine practice and long-term sustainability.

### 4.2. Strengths and Limitations

#### 4.2.1. Engagement and Acceptance by Interest-Holders

To enhance acceptability and thereby feasibility, interest-holder engagement was emphasised throughout the project. Nevertheless, the findings indicate that while the intervention was widely perceived as acceptable at the individual and professional levels, this alone was insufficient to ensure sustained enactment. Engagement was driven primarily by a small group of committed staff, whereas implementation in the broader home care organisation depended largely on managerial prioritisation and available resources. Organisational constraints, particularly time pressure and competing demands, limited leadership capacity to support implementation, thereby restricting the translation of staff acceptability into routine practice. These results underline that acceptability and motivation among practitioners must be matched by organisational support and continuous competence development if preventive interventions are to be sustained and aligned with evidence-based practice [101,102].

#### 4.2.2. Potential Harmful Consequences

Successful implementation and long-term sustainability depend on an intervention’s alignment with existing practices and infrastructures, which this study confirmed [37]. As Bonell et al. (2015) argue, public-health interventions may generate harmful consequences because they inevitably disrupt established routines and mechanism–context interactions [103]. To minimise such risks, the intervention was refined during the project to better fit current practice, and leaders further reduced potential harm by prioritising only selected components. This mitigated the risk that the intervention would divert time from more urgent tasks. However, following these adaptations, only weight monitoring is likely to be sustained. Although weight is a pragmatic and frequently used indicator [30], it reflects a symptom rather than an underlying risk factor [104]. Still, it represents incremental progress, and with stronger political support, further small but sustainable steps toward evidence-based nutritional practice may be achievable.

A second potential negative consequence concerns ‘false positives’, where individuals may be incorrectly identified as at risk [103]. Although the intervention was minimally invasive and focused on risk identification rather than diagnosis, case B3 illustrated the possibility of redundancy and the importance of recognising age-related health changes. This aligns with Rosendal et al. (2026), who found that some older adults resist interventions that challenge autonomy or when age-related decline feels more legitimate than expectations of ‘positive ageing’ [105]. Notably, B3 did not object to weighing or answering questions but expressed limited interest in making substantial lifestyle changes based on national recommendations.

#### 4.2.3. The Role of TL

A further limitation concerns TL’s dual role as both facilitator and evaluator. High levels of delivery and fidelity in Part 1 appeared to depend heavily on TL’s active facilitation, suggesting that feasibility under these conditions reflected supported rather than routine implementation. The decline observed in Part 2 indicates that the intervention was insufficiently integrated into everyday home care practice once this level of external facilitation was reduced. This pattern suggests that TL’s involvement may have temporarily compensated for organisational constraints such as time pressure and competing priorities, which became more visible in the absence of intensive support. This interpretation aligns with implementation research emphasising that externally driven implementation support can enhance early delivery, but is unlikely to sustain change unless internal facilitation capacity is established [106]. From an i-PARIHS perspective, the champions functioned as internal facilitators in the present study; however, their capacity to fully assume this role appeared insufficiently developed to sustain delivery independently. Accordingly, the feasibility findings should be interpreted with caution, as sustained enactment likely depends on deliberate strengthening of internal facilitation capacity alongside organisational support.

#### 4.2.4. Transferability and Interpretation of Findings

The transferability of the findings should be considered in relation to both participant selection and contextual conditions.

OAs were recruited through strategic selection in Part 1 and staff-mediated recruitment in Part 2. Consequently, participating OAs may have differed from non-participants in ways that influenced their engagement with the intervention. Furthermore, although perspectives from the wider staff group were captured through observations when the champions engaged their colleagues through learning sessions in Part 2, the qualitative evaluation relied primarily on data generated by a small group of highly engaged staff members (the champions) who played a central role throughout the feasibility study. Their experiences may therefore have influenced the interpretation of feasibility and acceptability. The findings should consequently be interpreted as context-dependent and their transferability to other home care settings should be considered in relation to local organisational conditions, resources, and staff cultures.

In addition, implementation and feasibility were influenced by multiple interacting contextual factors, including leadership priorities, organisational infrastructures, resource constraints, staff engagement, and variation in OAs’ health status and motivation. As these factors interacted with the intervention and its proposed mechanisms of change, the influence of individual contextual conditions cannot be fully disentangled. However, examining how such contextual conditions shaped intervention delivery, implementation, and mechanism activation was a central purpose of the feasibility study and embedded process evaluation.

## 5. Conclusions

This feasibility evaluation demonstrated that the intervention could be delivered in a real-world home care setting and was generally acceptable to both OAs and home care staff. However, delivery, implementation and activation of proposed mechanisms were constrained by barriers related to organisational conditions, work practices, workplace culture and OAs’ prerequisites for engagement. The findings informed refinements to the intervention and highlighted the need for further adaptation of both the intervention and its programme theory to improve fit with routine practice and support future scale-up. The findings also indicate that ongoing collaboration with municipal interest-holders may be important in addressing cultural and organisational factors, including leadership priorities, that influence implementation and sustained use of the intervention.

## Figures and Tables

**Table 1:** Overview of Research questions.

(RQ1) Delivery: To what extent were intervention activities delivered, and were they delivered as intended?
(RQ2) Key uncertainties: What factors mainly affect the delivery and implementation of the intervention?
(RQ3) Mechanisms: Were intended mechanisms activated for (a) home care staff and (b) the OAs—and what affected the activation? (a) Home care staff: competencies, skills, and knowledge gained via tailored learning activities. (b) OAs: nutritional self-care gained via knowledge and motivational interviewing.
(RQ4) Sustainability: How well sustained was the intervention, and what affected sustainability?

**Table 2:** Overview of activities in the intervention.

Part	Activities	Content and Short Description
Implementation strategy	A	An interprofessional workgroup with monthly or ad hoc meetings during the feasibility study. The group consisted of dietitians, selected home care staff, and the daily home care manager. The group had two aims: Serve as the reference group to enhance implementation [37]. Strengthen collaboration and clarify roles between dietitians and home care staff [24,38]. Additionally, home care staff from the group acted as ‘champions’ of the intervention and as mentors to their colleagues. The group was referred to as ‘the key group’ (detailed in the table in Section 2.5). Its members were volunteer permanent staff with an interest in nutrition who had been employed for at least one year, ensuring familiarity with current practices.
	B	Learning activities tailored to home care staff [24,38,39,40,41,42,43,44,45] and consisting of the following: I. E-learning modules: Four short videos (32 min in total), a PowerPoint presentation and a case-based learning activity. Led by mentors. II. Dietitian-led sessions: A ‘75 min session’, completed once per home care team during the feasibility study. Prepared by dietitians. III. Mentors: In Part 1, TL mentored staff from the key group. In Part 2, the key group acted as mentors to their colleagues.
Screening procedure	C	A start-up conversation with OAs and their informal caregivers [24]. Information about malnutrition risk, nutrition, and physical activity, provided both verbally and in a brochure. Introduction to the screening tool and the first use of the screening tool Motivational interviewing technique to encourage nutritional self-care and independent use of the tools [46,47]. Measurements: Weight, height, BMI, and calf circumference (as an indicator of muscle mass) were recorded. Only ‘weight’ is part of the screening tool (a), but BMI and calf circumference are relevant for diagnosing malnutrition [48,49] and were included to assess feasibility.
	D	Follow-up screenings: Monthly, continuous, and systematic screening using a screening tool, combined with an action guide outlining subsequent steps if the screening was positive [2,20,30,50,51]. Motivational interviewing for continued encouragement of nutritional self-care and independent use of the tools [46,47]. The screening tool consists of three parts: Unintended weight loss ≥ 1 kg/month (measured or self-reported). Risk factors via a 13-item questionnaire. Reduced physical activity, as this is an indicator for malnutrition risk [52]. Action guide: Positive screening triggers EVS (Eating Validation Scheme [53]). If EVS > 0, further action has to be taken.

**Table 4:** Dose, reach, and fidelity of interprofessional workgroup.

Parameter	Operationalisation	Measurements/Comments
Dose (delivered)	Planned number of meetings	13 (once a month)
	Meetings conducted	8/13 = 62%
Reach	Attendance by group members	Attendance as planned, except during annual leave or illness.
Fidelity (quality of delivery)	Delivery according to protocol	Members turned up when scheduled in their calendars.

**Table 5:** Dose, reach, and fidelity of learning activities.

Parameter	Operationalisation	Measurements/Comments
Dose (delivered)	I. E-learning sessions conducted	3/3 = 100%
	II. Dietitian-led sessions conducted	3/3 = 100%
	III. Mentor assistance	TL mentored key group members in Part 1. In Part 2, all home care staff were proposed mentor assistance.
Reach	I. Staff participation in e-learning sessions	Home care staff participated when attendance was scheduled as part of their work duties.
	II. Staff participation in dietitian-led sessions	Home care staff participated when attendance was scheduled as part of their work duties.
	III. Staff accepting mentor assistance	Part 1: All key group members (TL mentoring) Part 2: 3/16 = 19%
Fidelity (quality of delivery)	I. Delivery of e-learning sessions according to protocol	Two key group members lead the sessions for their colleagues as planned.
	II. Delivery of dietitian-led sessions according to protocol	Dietitians lead the sessions as planned.
	III. Delivery of mentor assistance according to protocol	Assistance was intended to match the specific needs of each staff member. This goal seems to have been met for individuals accepting mentor assistance.

**Table 6:** Dose, reach, and fidelity of start-up conversations.

Parameter	Operationalisation	Part 1	Part 2
Dose (delivered)	Full delivery (all components)	12/12 = 100% (TL mentoring)	Focus group interview with staff indicates selection in components, e.g., brochure not used, as the OA perceived sufficient nutritional knowledge.
Reach	OA chosen for participation	12	16 (9 not invited by staff)
	OAs accepting participation	12/12 = 100%	7 invited; 5 accepted; 5/7 = 71%
	Conversations conducted	12/12 = 100%	5/16 = 31%
Fidelity (quality of delivery)	Delivery according to protocol	Managerial reluctance limited the participation of a trained professional (e.g., dietitian). TL led the conversations.	Not applicable (only sparse notes in NEXUS)

**Table 7:** Dose, reach, and fidelity of follow-up screenings.

Parameter	Operationalisation	Part 1	Part 2
Dose (delivered)	Opportunity for screenings	12 OAs were followed → 71 possible screenings	26 OAs were followed → 50 possible screenings
	Screenings conducted	61/71 = 86%	37/50 = 74%
	Full delivery	36/71 = 51%	18/50 = 36%
	Partial delivery *	25/71 = 35%	19/50 = 38%
Reach	OAs receiving ≥1 screening visits	12/12 = 100%	4/14 = 29% (only ‘new’ OAs in Part 2 counted)
	OAs independent use of weight scheme (tool A)	0/12	0/14
	OAs measuring own weight	3/12 = 25%	Not observed
Fidelity (quality of delivery)	Delivery according to protocol	TL supported full delivery as planned by mentoring home care staff	Not applicable for ‘new’ staff members. Key group members were followed subsequently by TL, and observations indicate improved fidelity over time, but with differences among staff members.
	Documentation quality	TL supported documentation	Variation observed in NEXUS: some only record ‘no changes’; others provide full notes.

* Partial delivery refers to the measurement of weight alone, which was often unrelated to the intervention.

**Table 8:** Overview of Key Barriers and Their Influence on Delivery and Implementation.

Domain	Barrier Identified	Influence on Delivery and Implementation
**Organisational conditions**	Limited time allocation and competing priorities	Preventive nutritional care was frequently deprioritised in favour of urgent tasks
	Leadership hesitation regarding resource allocation	Reduced opportunity to deliver intervention activities as intended
	Lack of integration into NEXUS and existing infrastructures	Reduced feasibility and fit within routine practice
**Work practices**	Reliance on drivers’ lists and existing workflows	Delivery depended on explicit scheduling and reminders
	Limited interdisciplinary communication and collaboration	Constrained implementation and follow-up actions
**Workplace culture**	Predominance of reactive rather than preventive practice	Reduced prioritisation of screening activities
	Variable collective acceptance of the intervention	Reduced uptake among some staff members in Part 2
**Older adults’ prerequisites**	Poor health, fatigue, multimorbidity and cognitive limitations	Limited engagement and activation of nutritional self-care
	Established habits, social influences and low perceived relevance of prevention	Reduced motivation for behavioural change

## Data Availability

The original contributions presented in this study are included in the article. Further inquiries can be directed to the corresponding author.
